# Influence of Some Personal and Family Variables on Social Responsibility Among Primary Education Students

**DOI:** 10.3389/fpsyg.2020.01124

**Published:** 2020-06-12

**Authors:** Luis J. Martín-Antón, Miguel A. Carbonero, Juan A. Valdivieso, Eugenio Monsalvo

**Affiliations:** ^1^Excellence Research Group GR179 Educational Psychology, Department of Psychology, University of Valladolid, Valladolid, Spain; ^2^Junta de Castilla y León, Instituto de Enseñanza Secundaria Delicias (Ret.), Valladolid, Spain

**Keywords:** social responsibility, positive youth development, family nucleus, gender, primary school

## Abstract

One of the purposes of education is to help pupils develop a responsible attitude, which is understood to be the capacity to vouch for their actions appropriately and in a way that fits social norms. Training of this type should be intentional, planned, and personalized, which will depend on how developed the individual’s social responsibility is. This in turn is influenced by personal and family variables. This article provides an analysis of the interaction of some of those variables with the development of social responsibility in primary education pupils as the basis for the design of programs to promote personal and social responsibility tailored to the features of the pupils. To do this, the Social Responsibility Attitudes Scale was applied to 502 pupils taking grades 2 (8 years old), 4 (10 years old), and 6 (12 years old) of primary education. This scale measures the following factors: (a) obedient in family settings, (b) polite and accepting their mistakes, (c) trust in their parents, (d) responsibility in school setting, (e) friendly and willing to help, and (f) careful of the environment. By carrying out a multivariate analysis with the school grade, gender, family type (single, two-parent), and position among siblings (firstborn, only child, or not firstborn), it was concluded that attitudes related to prosociality start to be differentiated from grade 4 of primary education. It is in grade 6 that children become aware of their responsibility, and this is greater among that from two-parent families. However, no significant differences were found in the level of social responsibility with regard to gender or position among siblings.

## Introduction

Each society selects and implements the general value system that it considers appropriate to meet civic and social needs and demands, assigning a role of transmission and development to the school (Parra, 2003; Jukes et al., 2018). In this sense, owing to the social and value crises currently underway, school, the formation environments, must help to develop attitudes and promote citizens’ axiological values and knowledge (Mínguez, 2012, 2013). Thus, social responsibility is a relevant topic to develop in the school curriculum (Horner et al., 2010; Jones and Bouffard, 2012). It is part of the educational institution’s commitment to the groups involved: students, teachers, families, and environment (Severino-González et al., 2018). This is being now established institutionally in various countries, emphasizing the impact of educational centers in the community and in society in general (Larrán and Andrades, 2017; Martínez-Domínguez and Porto-Pedrosa, 2018) and expanding the importance and influence of schools in students’ moral development (Roofe, 2018).

Responsibility is considered as the ability to respond to one’s actions appropriately and effectively in accordance with the social norms (Keller, 2012). The teaching of this value should occur mainly in the educational and family spheres (Geboers et al., 2012), as it has a direct effect on academic performance and commitment (Carbonero et al., 2015) and on personal autonomy (Valero-Valenzuela et al., 2019). However, it should be implemented in everyday situations (Shiller, 2013), which should go beyond the private realm (Caba-Collado et al., 2016).

Several theories underpin social responsibility and from them are derived the operational variables in which we can intervene, including (a) the theory of reasoned action (Ajzen and Fishbein, 1974), which studies the interrelationship of subjective beliefs, attitudes, and rules when measuring the probability of a person’s intention to act; (b) the theory of symbolic interaction (Such and Walker, 2004; Beranek and Butler, 2006), which explains the formation of responsibility within people’s self-concept, based on the image they receive from others concerning honesty and justice, implying power and autonomy; (c) the theory of self-determination (Deci and Ryan, 2000), alluding to the need to develop the three basic psychological needs (competence, autonomy, and relationship with others), whose satisfaction increases personal well-being and intrinsic motivation; and d) the theory of positive action, which has generated intervention programs, taking as a starting point physical activity to improve the personal and social development of young people at risk of social exclusion (Escartí et al., 2006; Wright and Li, 2009; Escartí et al., 2010).

However, the systematic instruction of responsibility has not been a priority in general educational programs, although its positive effects are inferred (Barberá, 2001; Escámez and Gil, 2001; Alonso, 2002), given the importance and influence of the educational actions implemented in schools (Woitschach et al., 2017), as noted in programs applied in compulsory secondary education (Carbonero et al., 2017). The objectives and effectiveness of these programs are varied, although most of them have focused on increasing moral reasoning, attributions, self-concept, self-perception of efficacy, and comprehension of the world of others (Mestre et al., 1999; Barberá, 2001; Escámez and Gil, 2001; Hellison, 2003; Escartí et al., 2006; Lee and Martinek, 2009). The programs of Barberá (2001), Escámez and Gil (2001), and Monsalvo (2012, 2013) are the ones that focus the most on the academic aspects.

There have been more systematic and numerous examples of teaching social responsibility experiences by promoting responsibility in physical-sports activity (Belando et al., 2012; Richards et al., 2019) in various educational stages (Pavão et al., 2019). These programs have had very positive results (Ruiz et al., 2006; Caballero et al., 2013; Gordon and Doyle, 2015; Sánchez-Alcaraz et al., 2016; Andrew et al., 2018). The benefits go beyond an overall improvement in students’ social responsibility, also obtaining positive effects in the motivation, prosocial behaviors, reduction of violent behaviors, or improvements in the classroom climate (Manzano-Sánchez and Valero-Valenzuela, 2019). This can even be the basis of trauma-informed practice (TIP), aimed at ensuring the physical and emotional safety of disadvantaged students (Ellison et al., 2019). Particularly relevant are the studies that have implemented long-term programs, with large samples, using quantitative research methodology (Pozo et al., 2018). Of all of them, the one based on the *Teaching Personal and Social Responsibility* (TPRS) of Hellison’s (1995) model is one of the most consistent, starting with its instruction integrated into the curriculum, seeking generalization to other areas, and allowing student autonomy, on the basis of adequate interaction with the teacher, and a progressive increase in the level of responsibility.

Although from the viewpoint of educational psychology there has been no specific conceptual or methodological approach to this construct, it can be inferred from the differences in the components that the different proposed programs address. For example, the teaching of social and personal responsibility through TPRS differentiates between (Hellison, 2003; Hellison and Martinek, 2006; Escartí et al., 2011): (a) respecting rights and feelings; (b) participation and effort; (c) autonomy; (d) support; and (e) transfer. However, Alonso (2002) proposed the following altruistic components: (a) consistency with firm values (tolerance, generosity, etc.); (b) awareness of social needs and involvement in situations of injustice; (c) participation in improvement processes; and d) respect for assets of public interest.

Thus, it follows that there are several dimensions within the concept of social responsibility based on beliefs, attitudes, subjective rules, and behavioral intentions (Escámez and Gil, 2001; Monsalvo and Guaraná, 2008; Monsalvo, 2012, 2013):

1. *Polite and accepting their mistakes*, which associates the values of effort and self-management with personal well-being and individual development (Hellison, 2003). These aspects influence personal, social, and academic development, providing the necessary basis for adequate decision making on the basis of personal aspects, and not so influenced by the context (Rodríguez-Muñiz et al., 2019).
2. *Friendly and willing to help*, consistent with the values of development and social integration, associated with respect for the feelings and rights of others, and the ability to listen and put oneself in another’s place (Cecchini et al., 2003). When people can understand and behave according to these values, they have acquired *personal and social responsibility* (Hellison, 2003).
3. *Trust in their parents*. This dimension is essential for the development and consolidation of social responsibility as a civic–ethical and personal maturation component. The family is a space in which to enhance interpersonal relationships and to acquire the values of responsibility (Hammes et al., 2013; Mínguez, 2013).
4. *Obedience in the family setting*. This refers to the need to develop a full family socialization model to acquire and internalize values, principles, and rules related to prosocial behavior (Thomas, 2011).
5. *Responsibility in the school setting*. This is related to compliance with school rules, assumption of norms, and development of adequate attitudes toward the teaching-learning processes (Escámez et al., 2002; Gottfredson et al., 2004; Walsh et al., 2010; Regueiro et al., 2018).
6. *Careful of the environment.* This is linked to skills training for sustainable development and environmental care (Novo, 2009).

Teaching programs of personal and social responsibility share the development of respect, self-control, self-esteem, empathy, effort, autonomy, cooperation, helping others, and leadership (Sánchez-Alcaraz et al., 2016), in which the teacher’s role is essential (Hemphill et al., 2015; Lee and Choi, 2015). However, there are some discrepancies about the effectiveness of these programs, as their results are modulated by some variables. In this sense, greater effects have been observed in secondary education than in primary education (Sánchez-Alcaraz et al., 2012; Carbonero et al., 2017), although some authors point out that various components, such as respect or prosociality, are developed more in childhood than in adolescence (Hellison and Wright, 2003; Sánchez-Alcaraz et al., 2013). However, this development is conditioned by the characteristics of the socio-family environment (Zambrano et al., 2012), so the authors recommend applying these programs at an early age (Llopis-Goig et al., 2011). Moreover, teachers do not feel prepared to exercise their role as development drivers of social responsibility (Roofe, 2018; Ibaibarriaga-Toset and Tejero-González, 2020).

Gender is another variable that has been studied as a differentiator of levels of social responsibility. In this sense, higher general values have been found in males (Sánchez-Alcaraz et al., 2013), but as many of the studies have focused on the area of physical education, this trend may be modulated by the self-perception of physical competence (Moreno et al., 2009). In fact, other studies not related to physical activity indicate a higher level in females (Sosik et al., 2017; Buğdayci, 2019).

Finally, other authors consider it essential to involve the family environment in this learning process, incorporating the process into everyday life (Belando et al., 2012). Nuclear or single-parent families can determine the quality of the interaction at an early age (Olhaberry and Santelices, 2013), and children from bi-parent families obtain higher scores in variables such as communication and its relationship with life satisfaction (Dinisman et al., 2017; Camacho et al., 2020). However, it may depend more on the parents’ attitudes, conditioned by the socioeconomic situation of single-parent families than on the family structure itself (Mencarini et al., 2019).

## Aims of This Study

Consequently, in order to determine the selection of the components in which to intervene to promote social responsibility, this work analyses the influence of certain personal and family variables that may modulate the development of social responsibility in primary education: (a) the school grade: second grade (students aged 7–8), fourth grade (9–10 years), or sixth grade (11–12 years); (b) *gender*: male or female; (c) *family nucleus*: single-parent (living with a single parent) or bi-parent families (coexisting with both parents); and (d) *position of the siblings*: born-first child or only child or not firstborn child (the rest).

## Materials and Methods

### Participants

A total of 502 students (248 boys and 254 girls) from five public schools of primary education, enrolled in the second grade (*n* = 100, 19.9%), fourth grade (*n* = 212, 42.2%), and sixth grade (*n* = 190, 37.9%), aged between 7 and 12 years, participated in this study (*M* = 9.4, *SD* = 1.77). Regarding the family nucleus, most belong to a bi-parent family (*n* = 424, 84.5%), and the rest (*n* = 78, 15.5%) live in a single-parent family. As for the position of the siblings, there was the same number of firstborn children and only children (*n* = 251, 50%).

### Measures

*Assessment Scale of Social Responsibility Attitudes in Primary School* (EARSA-P, Monsalvo, 2013) is a questionnaire made up of 23 items rated on a Likert response scale ranging from 1 (*no difficulty*) to 4 (*very difficult*). The items are grouped into six factors with acceptable reliability coefficients: (F_1_) Obedience in the Family Setting (five items, α = 0.89); (F_2_) Polite and Accepting their mistakes (four items, α = 0.77); (F_3_) Trust in their Parents (four items, α = 0.86); (F_4_) Responsibility in the School Setting (five items, α = 0.81); (F_5_) Friendly and Willing to help (three items, α = 0.72); and (F_6_) Careful of the Environment (two items, α = 0.70). In this questionnaire, they were also asked to indicate their sociodemographic data such as gender, age, the grade in which they were enrolled, the composition of the family nucleus, number of siblings, and their position concerning their siblings, and the parents’ profession.

### Procedure

A letter requesting collaboration was sent to several schools explaining the reason and conditions of the study. Of the schools whose headmasters agreed to collaborate, five centers were selected at random, taking into account that all areas of the city were represented. Subsequently, the EARSA-P was applied to the students by their tutors, also incorporating a few previous questions about family aspects. It was applied orally to the second-year students, and the rest of them completed it collectively. Seventeen of the initial 519 participants (3.2%) were excluded from the final sample because they were absent at the time of data collection, mostly for health reasons.

### Data Analysis

After the parametric assumptions were checked, descriptive and inferential analyses were carried out, applying, in the first instance, a four-factor multivariate factorial design [multivariate analysis of variance (MANOVA)]. The independent variables were the aforementioned personal and family variables, and the dependent variables were the six social responsibility factors. The effect size was calculated using the partial eta square statistic, considering: 0.01 < η^2^*_*p*_* < 0.05, a small effect size; 0.06 < η^2^*_*p*_* < 0.13, a moderate effect size; and η^2^*_*p*_* > 0.14, a large effect size (Cohen, 1988). To examine in more depth the differences in the main effects, in the case of the variable Grade, we also performed a *post hoc* analysis with Scheffé’s test, as there were three non-homogeneous groups. In the rest of the variables, the parametric *t-*test for two independent groups was applied, also including the effect size by calculating Hedges’ *g* (Hedges, 1981; Hedges and Olkin, 1985), which, unlike Cohen’s *d*, takes into account the difference in the size of the groups, especially when using small samples. Cutoff points were established (Cohen, 1988, 1992): (a) *g* = 0.20, small effect size; (b) *g* = 0.50, moderate effect size; and (c) *g* = 0.80, large effect size. For cases where the parametric assumptions were not met, the Mann–Whitney *U*-test was used, calculating the non-parametric effect size using the *z* statistic (Rosenthal, 1991, 1994). The result was interpreted according to Cohen’s (1988) criterion: (a) *r* = 0.1, small effect size; (b) *r* = 0.3, moderate effect size, and (c) *r* = 0.5, large effect size. For this purpose, we used the statistical package IBM SPSS Statistics, version 26 (2019).

Previously, the validity of the EARSA-P was analyzed in the sample of this study, as it includes students of a lower age than the original validation. For this purpose, item factoring was calculated using the unweighted least squares (ULS) method, selecting components with eigenvalues greater than 1 and a promax oblique rotation method (Hendrickson and White, 1964), based on polychoric correlations, using the statistical software FACTOR v.10.10 (Ferrando and Lorenzo-Seva, 2017). To check the goodness of fit, we used Bentler’s simplicity index (Bentler, 1977) and the Satorra–Bentler scaled statistic (S-B χ^2^, *p* > *0.05*). However, as the goodness of fit is highly mediated by sample size, it was supplemented with other fit indexes (Bollen and Long, 1993; Hu and Bentler, 1999): the relative chi-square index (χ^2^/*df*) – whose value should be less than 2, but it is also considered acceptable if it is less than 5 (Schumacker and Lomax, 2004) – the comparative fit index (CFI > 0.95), the non-normed fit index (NNFI > 0.90), and the robust root mean-square error of approximation (RMSEA < 0.08).

All statistical analyses used showed a 95% confidence level.

## Results

### Psychometric Properties of the Assessment Scale of Social Responsibility Attitudes in Primary School

In our sample, this instrument explained 64% of the variance of social responsibility and presented adequate psychometric properties: Kaiser–Meyer–Olkin (KMO = 0.88), Bartlett’s sphericity test, χ^2^(253) = 5.092.0, *p* ≤ 0.001; S-B χ^2^(130) = 167.23, *p* = 0.015; S-B χ^2^/*df* = 1.29, CFI = 0.997, NNFI = 0.995, goodness-of-fit index (GFI) = 0.994, RMSEA = 0.024, 90% CI [0.014, 0.024], Bentler’s simplicity index *S* = 0.91 (P_100_), weighted root mean square residual (WRMR) = 0.028, and reliability coefficients between 0.74 and 0.86.

### Descriptive Analysis

As seen Table 1, there is a tendency to higher scores in all the social responsibility variables as the students’ grade advances, although that trend seems more acute between the second and third grades, especially in the factor Environmentally Caring. In contrast, there was an unnoticeable difference in the factor related to prosociality (Friendly and Willing to help). The same is true for all factors as a function of gender. As for the composition of the family nucleus, in all factors, children living with both parents obtained much higher scores. In contrast, concerning the position of the siblings, there were no differences, although the non-firstborn children always obtained slightly higher values.

**TABLE 1:** Distribution and descriptive statistics of the degree of social responsibility as a function of grade, gender, family nucleus, and position among siblings.

	**Total**	**Grade**			**Gender**		**Family nucleus**		**Position among siblings**	
		**2nd**	**4th**	**6th**	**M**	**F**	**S**	**TP**	**FB**	**NFB**
Frequency	502	100	212	190	248	254	424	78	251	251
Percentage	100	19.9	42.2	37.9	49.4	50.6	84.5	15.5	50	50
**OF**										
M	15.15	14.70	14.90	15.67	14.88	15.41	15.28	14.47	14.86	15.44
SD	3.39	3.34	3.42	3.33	3.33	3.43	3.31	3.73	3.40	3.36
**PAM**										
M	13.02	12.82	12.95	13.22	12.82	13.23	13.15	12.33	12.81	13.24
SD	2.47	2.57	2.53	2.34	2.45	2.48	2.40	2.73	2.56	2.36
**TP**										
M	11.62	11.07	11.63	11.91	11.30	11.94	11.85	10.43	11.39	11.86
SD	3.46	3.56	3.29	3.58	3.54	3.36	3.39	3.60	3.60	3.31
**RSS**										
M	17.48	17.33	17.23	17.85	17.20	17.74	17.63	16.50	17.35	17.61
SD	2.54	2.47	2.77	2.27	2.71	2.34	2.35	3.23	2.68	2.39
**FWH**										
M	10.23	10.01	10.19	10.38	10.21	10.24	10.36	9.50	10.19	10.26
SD	1.72	1.93	1.72	1.60	1.75	1.70	1.63	2.01	1.76	1.69
**CE**										
M	6.68	6.35	6.84	6.67	6.57	6.78	6.75	6.30	6.62	6.73
SD	1.52	1.76	1.39	1.48	1.52	1.51	1.46	1.74	1.52	1.51

**M, male; F, female; S, single-parent; TP, two-parent; FB, firstborn or only child; NFB, not firstborn; OF, obedient in family setting; PAM, polite and accepting their mistakes; TP, trust in their parents; RSS, responsibility in school setting; FWH, friendly and willing to help; CE, careful of the environment.**

### Multivariate Analyses

Using a 3 × 2 × 2 × 2 multivariate factor (MANOVA) analysis, we examined the effects of the interaction of the independent variables [(a) grade; (b) gender; (c) family nucleus; and (d) sibling position] on the target dependent variables. The MANOVA results (Table 2) revealed a single Grade × Family Nucleus interaction, ∧ = *0.951*, *F*(12, 948) = 1.77, *p* = 0.046, albeit with a low effect size (η^2^_*p*_ = 0.02). Precisely, there were statistically significant differences in the main effects of these two variables, with a moderate effect size in the case of the Family Nucleus, ∧ = *0.956, F*(6, 474) = 5.03, *p* < 0.001. η^2^*_*p*_* = 0.06, whereas in Grade, there was a low effect size, ∧ = *0.941, F*(12, 948) = 2.91, *p* = 0.001. η^2^*_*p*_* = 0.04.

**TABLE 2:** Multivariate analysis of variance (3^a^ × 2^b^ × 2^c^ × 2^d^).

	**∧**	***F***	***p***
Grade (A)	0.930	*F*(2,948) = 2.91	0.001
Gender (B)	0.992	*F* (6,474) = 0.60	0.731
Family nucleus (C)	0.940	*F* (6,474) = 5.03	<0.001
Position among siblings (D)	0.989	*F* (6,474) = 0.87	0.516
A × B	0.977	*F* (12,948) = 0.94	0.507
A × C	0.956	*F* (12,948) = 1.77	0.046
A × D	0.973	*F* (12,948) = 1.10	0.353
B × C	0.992	*F* (6,474) = 0.64	0.698
B × D	0.989	*F* (6,474) = 0.86	0.527
C × D	0.978	*F* (6,474) = 1.81	0.095
A × B × C	0.960	*F* (12,948) = 1.65	0.074
A × B × D	0.961	*F* (12,948) = 1.61	0.084
A × C × D	0.979	*F* (12,948) = 0.85	0.602
B × C × D	0.991	*F* (6,474) = 0.71	0.641
A × B × C × D	0.989	*F* (6,474) = 0.84	0.539

**^*a*^a_1_, second grade; a_2_, fourth grade; a_3_, sixth grade. ^*b*^b_1_, male; b_2_, female. ^*c*^c_1_, single-parent; c_2_, two-parent. ^*d*^c_1_, firstborn, only child; c_2_, not firstborn.**

### Main Effects of the Variable Grade

Statistically significant differences were yielded in two of the social responsibility factors (Table 3), with small effect sizes: (a) Friendly and Willing to help, *F*(2, 502) = 3.52, *p* = 0.030, η^2^*_*p*_* = 0.01; and (b) Environmentally Caring, *F*(2, 502) = 3.96, *p* = 0.020, η^2^*_*p*_* = 0.02. In both cases, students in the second grade had significantly lower scores than those in the fourth and sixth grades.

**TABLE 3:** Means (standard deviations), *F*-values, level of significance, and Scheffé test^*d*^ for the three grades in the dimensions of social responsibility.

**Social responsibility**	**Grade**				
	**Second grade^a^**	**Fourth grade^b^**	**Sixth grade^c^**	***F*(2, 502)**	***p***
Obedient in family setting	14.70 (3.34)	14.90 (3.42)	15.67 (3.33)	2.70	0.068
Polite and accepting their mistakes	12.82 (2.57)	12.95 (2.53)	13.22 (2.34)	0.12	0.880
Trust in their parents	11.07 (3.56)	11.63 (3.29)	11.91 (3.58)	1.41	0.245
Responsibility in school setting	17.33 (2.47)	17.23 (2.77)	17.85 (2.27)	2.27	0.104
Friendly and willing to help	10.01(1.93)^1^	10.19(1.72)^2^	10.38(1.60)^2^	3.52	0.030
Careful of the environment	6.35(1.76)^1^	6.84(1.39)^2^	6.67(1.48)^2^	3.96	0.020

**^*a*^n = 100. ^*b*^n = 212. ^*c*^n = 190.*^*d*^α = 0.05. ^1,2^Significant differences between groups (in which 1 < 2 < 3) [example: the second grade (1) group has a lower score than the other two. However, the fourth grade (2) and sixth grade (2) do not].*

### Main Effects of the Family Nucleus Variable

Table 4 shows that, albeit with low effect size, there were statistically significant differences in the social responsibility factors, except for Obedience in the Family Setting, *t*(500) = 1.93, *p* = 0.054. The variable Responsibility in the School Setting presented the highest effect size (*g* = 0.48).

**TABLE 4:** Means (standard deviations), *t*-value and level of significance, and effect size in the factors of social responsibility as a function of family nucleus.

**Social responsibility**	**Family nucleus**				
	**Single-parent^a^**	**Two-parent^b^**	***t*(500)**	***p***	***g***
Obedient in family setting	15.28 (3.31)	14.47 (3.73)	1.93	0.054	0.24
Polite and accepting their mistakes	13.15 (2.40)	12.33 (2.73)	2.72	0.007	0.33
Trust in their parents	11.86 (3.39)	10.43 (3.60)	3.33	0.001	0.42
Responsibility in school setting	20.94 (2.78)	19.55 (3.59)	3.03	< 0.001	0.48
Friendly and willing to help	16.89 (2.50)	15.92 (2.83)	3.58	0.002	0.38
Careful of the environment	6.75 (1.46)	6.30 (1.74)	2.10	0.018	0.30

**^*a*^n = 424. ^*b*^n = 78.**

### Effects of the Interaction of the Grade and Family Nucleus Variables

In a more detailed analysis of both variables (Table 5), we found that the differences occurred mainly in the fourth grade, in which the children living with both parents obtained significantly higher scores in all factors except for Polite and Accepting their mistakes, where there were no statistically significant group differences. However, in the second grade, there were only differences in Friendly and Willing to help, with higher scores in those living with both parents (*M* = 10.17, *SD* = 1.92) compared with those living in a single-parent nucleus (*M* = 8.92, *SD* = 1.71), *U* = 334.5, *p* = 0.015, with a low effect size (*r* = 0.02). In the sixth grade, the same variable Friendly and Willing to help showed significantly higher scores in children living in a bi-parent family nucleus (*M* = 10.49, *SD* = 1.51) than in those from single-parent families (*M* = 9.83, *SD* = 1.95), *t*(188) = 2.09, *p* = 0.038, with a moderate effect size (*g* = 0.41). However, in the sixth grade, there were also differences in the variable Polite and Accepting their mistakes, but always with higher scores in children living with both parents (*M* = 13.46, *SD* = 2.15) than in those living with only one parent (*M* = 12.03, *SD* = 2.94), *t*(188) = 3.17, *p* = 0.002, with a moderate effect size (*g* = 0.62).

**TABLE 5:** Means (standard deviations), *t*-value and level of significance, and effect size in the factors of social responsibility as a function of family nucleus in fourth grade.

**Social responsibility**	**Family nucleus**				
	**Single-parent^a^**	**Two-parent^b^**	***t*(210)**	***p***	***g***
Obedient in family setting	15.21 (3.28)	13.26 (3.72)	3.10	0.002	0.58
Polite and accepting their mistakes	13.09 (2.46)	12.23 (2.81)	1.81	0.071	0.34
Trust in their parents	11.93 (3.17)	10.09 (3.51)	3.04	0.003	0.57
Responsibility in school setting	17.48 (2.57)	15.85 (3.35)	3.23	0.001	0.60
Friendly and willing to help	10.34 (1.59)	9.41 (2.16)	2.94	0.004	0.55
Careful of the environment	6.93 (1.36)	6.38 (1.46)	2.13	0.034	0.40

**^*a*^n = 178. ^*b*^n = 34.**

## Discussion

It has been shown that there are no differences as a function of gender and sibling position in social responsibility. However, it is conclusive that attitudes of prosociality (Friendly and Willing to help and Environmentally Caring) are different in the second grade from the rest of the stages, indicating a progressive development of awareness of responsibility, although not on a constant basis (Buğdayci, 2019). These results coincide with those obtained in the application of the theory of young people’s positive development of personal and social responsibility (Cecchini et al., 2003; Gutiérrez et al., 2011), which, in general, are very influential for positive social development (Balderson and Sharpe, 2005; Caba-Collado et al., 2016).

The variable composition of the Family Nucleus produced the most differences in attitudes of responsibility. Children living with both parents obtained higher levels in all the dimensions, except for Obedience in the Family Setting, indicating a modulating effect of the socio-family environment on the development of social responsibility (Sosik et al., 2017). However, these results are not consistent across all grades, because in the fourth grade, there was more disruption, which was subsequently reduced. In short, it seems that social responsibility and the progressive development of moral autonomy are linked (Osler and Starkey, 2005), and research with adolescents has found positive and predictive relationships between them (Moreno et al., 2009; Gutiérrez et al., 2011). Responsibility programs play a fundamental role in the development of autonomy (Ellison et al., 2019; Valero-Valenzuela et al., 2019); contributing to the improvement of cognitive skills (Jukes et al., 2018); the perception of competence and intrinsic motivation (Manzano-Sánchez and Valero-Valenzuela, 2019); fun, satisfaction with life, empathy, and prosocial behavior (Cecchini et al., 2003; Moreno et al., 2009; Gutiérrez et al., 2011); and education in values in general (Sánchez-Alcaraz et al., 2016). When the community is involved, these programs also help to minimize aggressive behavior (Lorenzo-Seva et al., 2010) and improve contexts other than the context in which the program was implemented (Caballero et al., 2013).

In this work, we observe that the binomial civil social responsibility–autonomy begins to be acquired in the sixth grade, when children become aware of their attitudes of prosociality and caring for the environment. They still continue to understand “being responsible” as being obedient in the school and family settings, which coincides with the conclusions of Such and Walker (2004), for whom, from children’s perspective, “doing things responsibly,” that is, with wisdom, maturity, and trust, is not the same thing as “doing responsible things.” In this case, acting responsibly allows them access to more responsible situations (choosing when and how to do tasks, staying alone at home, etc.), which, for many of them, implies a greater degree of autonomy. In this line, reaffirming this conclusion, this forces us to accept the child as a responsible being, within the social community where he or she lives (Lister, 2008). However, this acceptance is partly due to children’s proving their ability to do things responsibly (Escartí et al., 2012). For adults, bringing together both statements has led other authors (Osler and Starkey, 2005; Ochs and Izquierdo, 2009; Thomas, 2011) to approaches that could be framed within the self-determination theory (Deci and Ryan, 2000), which would include the acquisition of attitudes of responsibility in didactic work (Alonso, 2002; Monsalvo and Guaraná, 2008; Monsalvo, 2012, 2013; Carbonero et al., 2017; Andrew et al., 2018).

## Conclusion

This work deepens the differential analysis of the degree of social responsibility on the basis of some variables that can condition its development. In this way, schools can design and implement concrete educational actions to promote specific dimensions of social responsibility that the students may be lacking or that may be more beneficial, considering the children’s evolutionary stage. In this sense, gender is not a differentiating variable, nor is the child’s position in relation to the siblings or being an only child. However, the higher scores in most of the social responsibility dimensions in children living in bi-parent families than in children living in single-parent families were noteworthy. However, this may be determined more by the socioeconomic situation than by the family structure itself. On the other hand, it seems that the intermediate grades in primary education mark a turning point in the development of moral autonomy, as children may be more aware of the actions involved in being responsible, and not so dependent on family guidance. As a result, around the fourth grade of primary education may be a key moment for the implementation of specific programs for the development of social responsibility.

In any case, it should be considered that this study was carried out considering variables that are constantly evolving socially. The emerging diversity of the different types of family structure is greatly conditioned by other cultural and social aspects, which go beyond the educational context. The two most frequent family core systems were taken into account, but we should not forget other types of family structure that may be camouflaged within the single-parent category and, owing to their frequency, could be also be studied.

In conclusion, the results present a didactic challenge, as systematic programs to promote social responsibility must enhance student autonomy, understood at this stage as prosociality and environmentally caring. In later stages, autonomy is also associated with emotional well-being (Amérigo et al., 2013) and shows positive effects on students’ social development. The following could also be studied: (a) variables related to personal and social responsibility in early child education, such as the influence that the adult model has on children’s development, especially the influence of parents and teachers; (b) concrete educational actions that may be the basis of specific programs to promote responsibility at an early age; and (c) the evolution of social and personal responsibility through longitudinal studies.

## Data Availability Statement

The datasets analyzed in this article are not publicly available because ethics approval was not sought or granted for such use. Requests to access the datasets should be directed to corresponding author (LM-A).

## Ethics Statement

The study was conducted in accordance with the 1964 Declaration of Helsinki and its later amendments or comparable ethical standards, with the approval of the management boards of the schools. Ethical approval was not required for this study in accordance with the national and institutional requirements. Participation in the study was voluntary. Written informed consent was obtained from the parents/legal guardians of all participants.

## Author Contributions

All authors made substantial contribution to the theoretical framework, design, data collection, interpretation of this study, and approved the final manuscript as submitted.

## Conflict of Interest

The authors declare that the research was conducted in the absence of any commercial or financial relationships that could be construed as a potential conflict of interest.
